# A Longitudinal Multicenter Study Comparing the Effectiveness of Pharmacological and Lifestyle-Only Interventions in Adults With Prediabetes for Preventing Acute Hyperglycemic Crises and Progression to Type 2 Diabetes Mellitus

**DOI:** 10.7759/cureus.95420

**Published:** 2025-10-26

**Authors:** Tauseef Ullah Shah, Talha Masood, M Hassaan Shah, Sibte Abbas, Fatima Tu Zuhra, Iqra Khalil, Muhammad Hamza Ghufran, Hamza Usman, Sundas Safdar, Sheema Iqbal, Naqeeb Ullah

**Affiliations:** 1 General Medicine, Khyber Teaching Hospital, Peshawar, PAK; 2 General Medicine, Northern Lincolnshire and Goole NHS Foundation Trust, Scunthorpe, GBR; 3 General Medicine, Al Bassam Medical Center, Neom, SAU; 4 Internal Medicine, Hayatabad Medical Complex, Peshawar, PAK; 5 Pediatrics, Ziauddin Hospital, Karachi, PAK; 6 Internal Medicine (Medical D Ward), Lady Reading Hospital, Peshawar, PAK; 7 Radiology, Lady Reading Hospital, Peshawar, PAK; 8 Public Health, Basic Health Unit (BHU) Chakdara, Lower Dir, PAK; 9 Internal Medicine, Lady Reading Hospital, Peshawar, PAK

**Keywords:** hyperglycemic crises, lifestyle modification, metformin, pharmacological intervention, prediabetes, type 2 diabetes mellitus

## Abstract

Background: Prediabetes is a high-risk metabolic state that, if not effectively managed, can progress to type 2 diabetes mellitus (T2DM) or acute hyperglycemic crises. Early intervention is, therefore, critical to reduce the burden of diabetes-related morbidity.

Objectives: The primary objective of this study was to compare the long-term effectiveness of pharmacological therapy vs. lifestyle-only interventions in preventing the progression of prediabetes to T2DM. The secondary objectives were to assess the incidence of acute hyperglycemic crises, as well as the long-term adherence and metabolic improvements associated with each intervention strategy.

Methods: This multicenter, longitudinal cohort study was conducted at Lady Reading Hospital and Khyber Teaching Hospital in Peshawar from January 2022 to December 2024. A total of 1,520 adults with prediabetes (defined as fasting plasma glucose 100-125 mg/dL or glycated hemoglobin, HbA1c, 5.7%-6.4%) were enrolled and followed for 24 months. Participants were assigned to either a pharmacological intervention group (n = 760) receiving metformin (500-1,000 mg twice daily) or an alternative agent when contraindicated, alongside standard counseling, or a lifestyle-only group (n = 760) following a structured program including individualized dietary counseling, at least 150 minutes of moderate physical activity per week, and monthly follow-up sessions for motivation and monitoring. Exclusion criteria included known diabetes, severe renal or hepatic impairment, pregnancy, or current use of glucose-lowering agents. Pill counts and follow-up attendance records assessed adherence. Anthropometric parameters, glycemic indices, lipid profiles, and blood pressure were measured at baseline and at each six-month interval. The primary outcomes were progression to T2DM and occurrence of acute hyperglycemic crises. Secondary outcomes included adherence rates and adverse events. Statistical analyses were performed using IBM Statistical Package for the Social Sciences software (version 26). Repeated measures analysis of variance, Kaplan-Meier survival curves, and Cox proportional hazards regression were used to evaluate predictors of progression, with p < 0.05 considered significant.

Results: Both intervention groups demonstrated significant improvements in weight, body mass index, fasting plasma glucose, HbA1c, and lipid parameters over 24 months (p < 0.001 for all). Adherence was higher in the pharmacological group: 655 (86.1%) vs. 598 (78.7%); p = 0.004. Progression to T2DM occurred in 72 (9.47%) patients in the pharmacological group and 94 (12.37%) in the lifestyle-only group (p = 0.03). Acute hyperglycemic crises were rare: six (0.79%) vs. seven (0.92%), p > 0.05. The composite outcome of progression to T2DM or hyperglycemic crises was significantly lower in the pharmacological group: 78 (10.26%) vs. 101 (13.29%), p = 0.02. Adverse events were mild and manageable, primarily gastrointestinal intolerance.

Conclusion: Both pharmacological and lifestyle interventions effectively improved metabolic outcomes among adults with prediabetes. However, pharmacological therapy provided modestly greater protection against disease progression and relapse. While the findings highlight the clinical value of early pharmacologic intervention, results should be interpreted with consideration of adherence variability and potential center-level differences. Larger randomized studies are warranted to confirm these observations.

## Introduction

Prediabetes represents an intermediate state of glucose dysregulation in which blood glucose levels are higher than normal but not yet meeting diagnostic criteria for type 2 diabetes mellitus (T2DM) [1]. This metabolic state is characterized by insulin resistance, impaired fasting glucose, and/or impaired glucose tolerance, and is widely recognized as a high-risk condition for progression to overt diabetes [2]. Globally, the prevalence of prediabetes has risen substantially over recent decades, driven by sedentary lifestyles, dietary transitions, urbanization, and the increasing burden of obesity [3]. Epidemiological evidence suggests that individuals with prediabetes not only face an elevated risk of developing T2DM but are also susceptible to acute hyperglycemic crises, including hyperosmolar hyperglycemic state (HHS) and, less commonly, diabetic ketoacidosis (DKA), even before a formal diabetes diagnosis [4,5].

Interventions targeting this stage have the potential to delay or prevent disease progression and avert acute complications [6]. Current preventive strategies typically fall into two broad categories: pharmacological therapy, such as metformin or other glucose-lowering agents, and lifestyle modification programs emphasizing dietary changes, increased physical activity, and weight reduction [7]. The American Diabetes Association (ADA) and other international bodies recommend both approaches, but optimal strategy selection remains debated, particularly in resource-diverse and ethnically varied populations [8].

Lifestyle modification is widely advocated as the cornerstone of diabetes prevention; however, adherence to behavioral interventions is often challenging in real-world settings, and their long-term sustainability is uncertain [9]. Pharmacological therapy, on the other hand, can achieve measurable glycemic benefits, but questions persist regarding cost-effectiveness, potential side effects, and patient acceptability when prescribed in the prediabetic stage [10]. Despite numerous studies, there are limited multicenter longitudinal data directly comparing these interventions in terms of preventing both acute hyperglycemic crises and progression to T2DM [11,12].

Understanding how these interventions perform over time in diverse populations is essential for developing effective, evidence-based preventive strategies. Therefore, this multicenter longitudinal cohort study was designed to provide real-world insights into their comparative effectiveness. The primary objective of this study was to compare the long-term effectiveness of pharmacological therapy vs. lifestyle-only interventions in preventing the progression of prediabetes to T2DM. The secondary objectives were to assess 1) the incidence of acute hyperglycemic crises, including DKA and HHS, and 2) adherence and metabolic improvements associated with each intervention strategy over a 24-month follow-up period.

## Materials and methods

Study design and setting

This multicenter, longitudinal, comparative cohort study was conducted at Lady Reading Hospital and Khyber Teaching Hospital (KTH), Peshawar, Pakistan. The study spanned three years, from January 2022 to December 2024, with each participant followed prospectively for 24 months from the time of enrollment.

Ethical approval

Ethical approval was obtained from the Institutional Review Board of KTH, Peshawar (approval no. 903/DOC/KMC, dated December 24, 2021). Written informed consent was obtained from all participants before recruitment. The study was conducted in accordance with the ethical principles outlined in the Declaration of Helsinki.

Inclusion and exclusion criteria

Adults aged 18-65 years who met the ADA diagnostic criteria for prediabetes were eligible for inclusion. Prediabetes was defined as impaired fasting glucose (fasting plasma glucose 100-125 mg/dL), impaired glucose tolerance (two-hour OGTT plasma glucose 140-199 mg/dL), or glycated hemoglobin (HbA1c) between 5.7% and 6.4%. All participants were required to provide informed consent and demonstrate willingness to comply with scheduled follow-up visits. Individuals were excluded if they had a prior diagnosis of T2DM, were pregnant or lactating, had contraindications to metformin such as severe renal impairment, suffered from comorbid conditions that limited life expectancy, or were unable to adhere to the follow-up or treatment protocol.

Sampling technique and sample size

A convenience sampling approach was employed to recruit eligible participants from the outpatient clinics of both participating centers. Over the three-year recruitment period, 1,680 adults meeting the inclusion criteria were enrolled. After accounting for losses to follow-up, withdrawals, and relocations, 1,520 participants successfully completed the 24-month follow-up and were included in the final analysis, with an equal distribution between the pharmacological intervention group (n = 760) and the lifestyle-only group (n = 760).

The sample size was determined through a priori power analysis informed by evidence from landmark diabetes prevention trials. The Diabetes Prevention Program (DPP) reported a 58% reduction in diabetes incidence with lifestyle intervention and a 31% reduction with metformin compared to placebo over 2.8 years, demonstrating a clinically meaningful absolute difference between strategies [13]. Similarly, the Da Qing Diabetes Prevention Study found that lifestyle modification delayed the onset of type 2 diabetes by approximately four years and reduced both cardiovascular and all-cause mortality [14]. In resource-limited South Asian contexts, smaller scale studies, such as a Nepalese cost-of-illness analysis involving only 142 participants, have yielded significant outcomes, supporting the feasibility and relevance of large observational cohorts in low- and middle-income countries [15].

Based on these data, we assumed a 15% progression rate to T2DM in the lifestyle-only group and a 9% rate in the pharmacological group (an absolute difference of 6%) over 24 months. Using a two-sided α = 0.05, 80% power, and the log-rank test, a minimum of 684 participants per arm (1,368 total) was required. Allowing for an anticipated 10% attrition rate, the target enrollment was set at 1,680 participants. The final cohort of 1,520 participants provided more than 85% statistical power to detect the hypothesized difference in diabetes progression between the two intervention strategies, ensuring robustness and generalizability of results.

Group allocation

**Figure 1:**
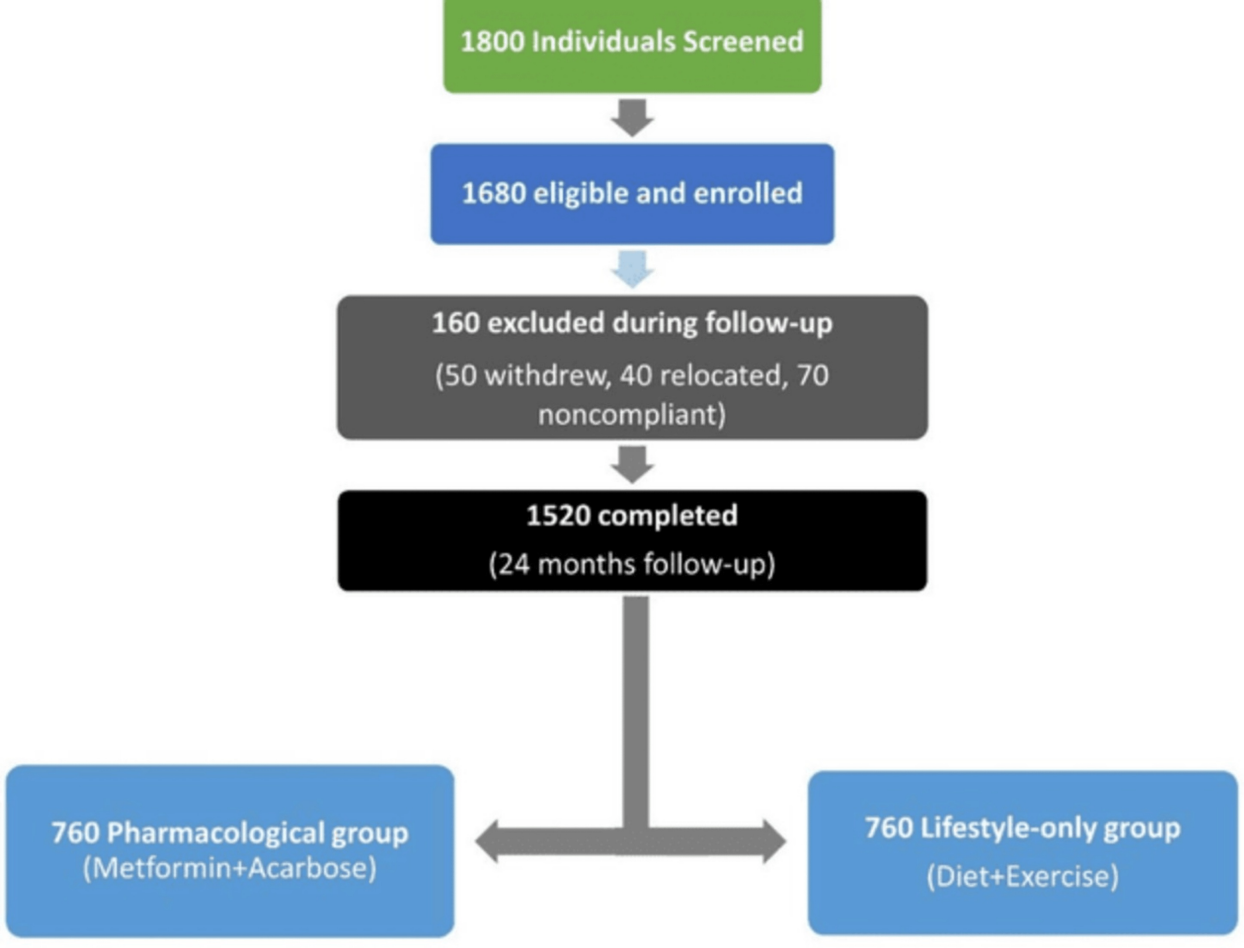
Study flow diagram

Participants were categorized into two intervention groups based on the assigned treatment strategy (Figure 1). The pharmacological intervention group received metformin in accordance with the U.S. Food and Drug Administration prescribing guidelines, initiated at 500 mg once daily, and titrated as tolerated up to a maximum of 2,000 mg per day. Participants unable to tolerate metformin were prescribed alternative pharmacological agents, such as acarbose, and these were recorded. The lifestyle-only group participated in a structured lifestyle modification program that included individualized dietary counseling, caloric moderation, improved nutritional balance, and engagement in at least 150 minutes per week of moderate-intensity physical activity. Participants were encouraged to achieve a 5%-7% reduction in baseline body weight, and reinforcement and counseling were provided at each follow-up visit.

Baseline assessment

At enrollment, baseline data collection included demographic details, medical and family history of diabetes, socioeconomic status, and dietary patterns. Anthropometric measurements such as weight, height, body mass index (BMI), waist circumference, and blood pressure were recorded. Laboratory investigations comprised fasting plasma glucose, oral glucose tolerance test (OGTT; where applicable), HbA1c, lipid profile, serum creatinine, and liver function tests. Relevant comorbidities were also documented to assess their potential influence on outcomes.

Follow-up and data collection

Participants were followed up at 6, 12, 18, and 24 months after enrollment. At each visit, adherence to the assigned intervention, occurrence of adverse events, and any new diagnosis of T2DM or acute hyperglycemic crisis were recorded. Anthropometric and laboratory parameters, including fasting plasma glucose, HbA1c, and lipid profile, were reassessed according to the study protocol. Although all visits were analyzed statistically, for clarity, only baseline and 24-month data are presented in the main results tables.

Outcome measures

The primary outcome was a composite of progression from prediabetes to T2DM, defined by ADA diagnostic thresholds, and/or the occurrence of acute hyperglycemic crisis during follow-up. Secondary outcomes included the individual event rates of diabetes progression and hyperglycemic crises, as well as longitudinal changes in HbA1c, fasting glucose, lipid parameters, BMI, and blood pressure. Adherence to assigned interventions was analyzed as a key behavioral outcome.

Data management and quality control

All data were entered into standardized electronic case report forms and stored in a password-protected database accessible only to study personnel. Site investigators were trained to ensure uniform protocol adherence, and periodic data verification and random audits were conducted to maintain quality control and minimize data entry errors.

Statistical analysis

All statistical analyses were performed using IBM Statistical Package for the Social Sciences Statistics version 26 (IBM Corp., Armonk, NY). Continuous variables were expressed as mean ± standard deviation or median with interquartile range, depending on data distribution, while categorical variables were summarized as frequencies and percentages. Between-group comparisons at baseline were performed using the independent t-test or Mann-Whitney U test for continuous variables and the chi-square test for categorical variables. Within-group longitudinal changes in metabolic and anthropometric parameters were analyzed using repeated measures analysis of variance. Adherence rates at 24 months were compared using the chi-square test. Time-to-event analyses for progression to T2DM, hyperglycemic crises, and the composite outcome were conducted using Kaplan-Meier survival curves and compared via the log-rank test. Cox proportional hazard regression models were applied to estimate hazard ratios (HRs) with 95% confidence intervals (CIs), adjusting for potential confounders such as age, sex, baseline BMI, HbA1c, socioeconomic status, comorbidities, and dietary patterns. A p value of <0.05 was considered statistically significant.

## Results

At baseline, the pharmacological (n = 760) and lifestyle-only (n = 760) groups were comparable in age (48.32 ± 9.45 vs. 47.89 ± 9.78 years); gender distribution: male, 398 (52.37%) vs. 405 (53.29%); and socioeconomic status (Table 1). Family history of diabetes was present in 438 (57.63%) vs. 430 (56.58%), while comorbidities such as hypertension, 152 (20.00%) vs. 145 (19.08%), and dyslipidemia, 198 (26.05%) vs. 203 (26.71%), were balanced. Dietary patterns were similarly distributed: healthy 212 (27.89%) vs. 218 (28.68%); unhealthy 150 (19.74%) each. No statistically significant differences were observed at baseline (all p > 0.05), indicating successful group comparability.

**Table 1:** Baseline demographic and clinical characteristics of study participants Values are presented as n (%) unless otherwise specified. Statistical significance was considered at p < 0.05. Chi-square test and independent t-test were applied as appropriate SD: standard deviation

Category	Characteristic	Pharmacological group (n = 760)	Lifestyle-only group (n = 760)	p value
Age, years, mean ± SD		48.32 ± 9.45	47.89 ± 9.78	0.42 (independent t-test)
Gender	Male	398 (52.37%)	405 (53.29%)	0.74 (chi-square test)
	Female	362 (47.63%)	355 (46.71%)	0.74 (chi-square test)
Socioeconomic status	Low	182 (23.95%)	176 (23.16%)	0.62 (chi-square test)
	Middle	412 (54.21%)	425 (55.92%)	
	High	166 (21.84%)	159 (20.92%)	
Family history	Diabetes	438 (57.63%)	430 (56.58%)	0.68 (chi-square test)
Comorbidities	Hypertension	152 (20.00%)	145 (19.08%)	0.61 (chi-square test)
	Dyslipidemia	198 (26.05%)	203 (26.71%)	0.80 (chi-square test)
Dietary pattern	Healthy	212 (27.89%)	218 (28.68%)	0.84 (chi-square test)
	Mixed	398 (52.37%)	392 (51.58%)	
	Unhealthy	150 (19.74%)	150 (19.74%)	

Both groups had similar baseline anthropometrics, with weight (79.45 ± 12.32 vs. 78.88 ± 11.95 kg), BMI (28.18 ± 3.91 vs. 27.87 ± 3.85 kg/m²), and waist circumference (98.56 ± 10.12 vs. 97.89 ± 9.98 cm), as shown in Table 2. Blood pressure readings were also comparable (systolic: 128.4 ± 14.6 vs. 127.9 ± 15.1 mmHg; diastolic: 81.2 ± 9.8 vs. 80.9 ± 9.6 mmHg). No significant differences were observed between the groups (all p > 0.05), confirming balanced baseline characteristics.

**Table 2:** Baseline anthropometric and clinical measurements Values are presented as mean ± standard deviation unless otherwise noted. Statistical significance was considered at p < 0.05. Independent t-test was used for comparisons BMI: body mass index; BP: blood pressure

Category	Test/parameter	Pharmacological group (n = 760)	Lifestyle-only group (n = 760)	p value	Statistical test
Anthropometrics	Weight, kg	79.45 ± 12.32	78.88 ± 11.95	0.45	Independent t-test
	Height, cm	167.8 ± 8.2	168.1 ± 7.9	0.52	
	BMI, kg/m²	28.18 ± 3.91	27.87 ± 3.85	0.12	
	Waist circumference, cm	98.56 ± 10.12	97.89 ± 9.98	0.21	
Blood pressure	Systolic BP, mmHg	128.4 ± 14.6	127.9 ± 15.1	0.42	
	Diastolic BP, mmHg	81.2 ± 9.8	80.9 ± 9.6	0.55	

Baseline glycemic and lipid profiles were similar, with fasting plasma glucose (108.4 ± 8.9 vs. 107.9 ± 9.1 mg/dL), 2-hour OGTT (152.6 ± 16.3 vs. 151.8 ± 15.9 mg/dL), and HbA1c (5.92 ± 0.21% vs. 5.90 ± 0.20%), as shown in Table 3. Total cholesterol (198.4 ± 32.5 vs. 196.7 ± 31.8 mg/dL), low-density lipoprotein, high-density lipoprotein, and triglycerides also showed no significant differences (all p > 0.05), supporting baseline equivalence of metabolic parameters.

**Table 3:** Baseline laboratory investigations Values are presented as mean ± standard deviation unless otherwise noted. Statistical significance was considered at p < 0.05. Independent t-test was used for comparisons OGTT: oral glucose tolerance test; HbA1c: glycated hemoglobin; LDL: low-density lipoprotein; HDL: high-density lipoprotein

Category	Test/parameter	Pharmacological group (n = 760)	Lifestyle-only group (n = 760)	p value	Statistical test
Glycemic parameters	Fasting plasma glucose, mg/dL	108.4 ± 8.9	107.9 ± 9.1	0.33	Independent t-test
	2-hour OGTT, mg/dL	152.6 ± 16.3	151.8 ± 15.9	0.46	
	HbA1c	5.92 ± 0.21	5.90 ± 0.20	0.28	
Lipid profile	Total cholesterol, mg/dL	198.4 ± 32.5	196.7 ± 31.8	0.25	
	LDL, mg/dL	126.2 ± 28.1	124.8 ± 27.5	0.31	
	HDL, mg/dL	44.8 ± 10.2	45.3 ± 9.8	0.48	
	Triglycerides, mg/dL	172.5 ± 49.3	170.8 ± 48.7	0.42	

**Figure 2:**
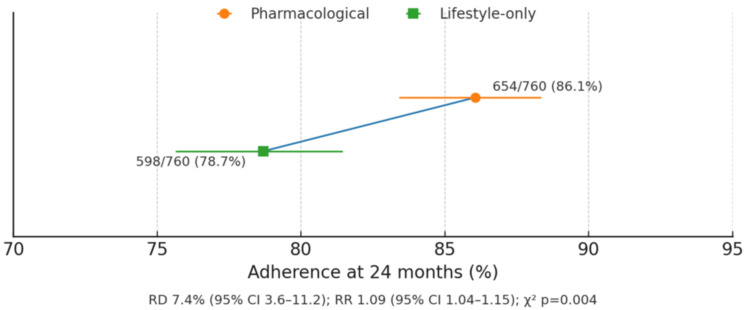
Adherence at 24 months by intervention Values are presented as mean ± standard deviation unless otherwise noted. Statistical significance was considered at p < 0.05. An independent t-test was used for comparisons RD: risk difference; RR: relative risk; CI: confidence interval

Overall adherence at 24 months was higher in the pharmacological group (86.05% vs. 78.68%, p = 0.004), reflecting better compliance with medication compared to lifestyle-only interventions. This likely contributed to observed differences in glycemic outcomes and highlights challenges in sustaining behavioral interventions over long-term follow-up (Figure 2).

Significant reductions in weight and BMI were observed in both intervention groups over 24 months (Table 4). In the pharmacological group, mean weight decreased from 79.45 kg at baseline to 75.17 kg at 24 months, and BMI declined from 28.18 to 26.83 kg/m² (p < 0.001). Similarly, the lifestyle-only group experienced weight reduction from 78.88 to 74.22 kg, and BMI decreased from 27.87 to 26.27 kg/m² (p < 0.001). These findings indicate that both pharmacological and lifestyle interventions effectively improved anthropometric outcomes in prediabetic adults.

**Table 4:** Anthropometric measurements at baseline and 24 months BMI: body mass index; ANOVA: analysis of variance

Category	Test/parameter	Baseline	24 months	p value	Statistical test
Anthropometrics	Weight, kg (pharmacological)	79.45	75.17	<0.001	Repeated measures ANOVA
	Weight, kg (lifestyle)	78.88	74.22	<0.001	
	BMI, kg/m² (pharmacological)	28.18	26.83	<0.001	
	BMI, kg/m² (lifestyle)	27.87	26.27	<0.001	

Both intervention groups showed substantial improvements in glycemic and lipid parameters over 24 months (Table 5). Fasting plasma glucose decreased from 108.4 to 99.8 mg/dL in the pharmacological group and from 107.9 to 100.8 mg/dL in the lifestyle group (p < 0.001). HbA1c declined from 5.92% to 5.55% vs. from 5.90% to 5.60% in the pharmacological and lifestyle groups, respectively (p < 0.001). Total cholesterol also improved significantly, falling from 198.4 to 184.9 mg/dL in the pharmacological group and from 196.7 to 183.8 mg/dL in the lifestyle group (p < 0.001). These results demonstrate effective metabolic control achieved by both intervention strategies.

**Table 5:** Laboratory parameters at baseline and 24 months (n = 1,520) FPG: fasting plasma glucose; HbA1c: glycated hemoglobin; ANOVA: analysis of variance

Category	Test/parameter	Baseline	24 months	p value	Statistical test
Glycemic parameters	FPG, mg/dL (pharmacological)	108.4	99.8	<0.001	Repeated measures ANOVA
	FPG, mg/dL (lifestyle)	107.9	100.8	<0.001	
	HbA1c, % (pharmacological)	5.92	5.55	<0.001	
	HbA1c, % (lifestyle)	5.90	5.60	<0.001	
Lipid profile	Total cholesterol, mg/dL (pharmacological)	198.4	184.9	<0.001	
	Total cholesterol, mg/dL (lifestyle)	196.7	183.8	<0.001	

Mean reductions in HbA1c (-0.37% vs. -0.30%, p = 0.04) and fasting plasma glucose (-8.6 vs. -7.1 mg/dL, p = 0.03) favored pharmacological intervention, while changes in BMI (-1.35 vs. -1.60 kg/m²), weight (-3.75 vs. -4.65 kg), total cholesterol (-13.5 vs. -12.9 mg/dL), and blood pressure were not statistically different, suggesting modest but significant glycemic benefits with pharmacotherapy (Table 6).

**Table 6:** Secondary outcomes: mean changes in key parameters from baseline to 24 months by intervention group HbA1c: glycated hemoglobin; FPG: fasting plasma glucose; BMI: body mass index; BP: blood pressure

Category	Parameter	Pharmacological Δ	Lifestyle Δ	p value	Statistical test
Glycemic parameters	HbA1c, %	-0.37	-0.30	0.04	Mann-Whitney U test
	FPG, mg/dL	-8.6	-7.1	0.03	
Anthropometrics	BMI, kg/m²	-1.35	-1.60	0.12	
	Weight, kg	-3.75	-4.65	0.08	
Lipid profile	Total cholesterol, mg/dL	-13.5	-12.9	0.51	
Blood pressure	Systolic BP, mmHg	-4.2	-3.9	0.62	
	Diastolic BP, mmHg	-2.1	-2.0	0.78	

**Figure 3:**
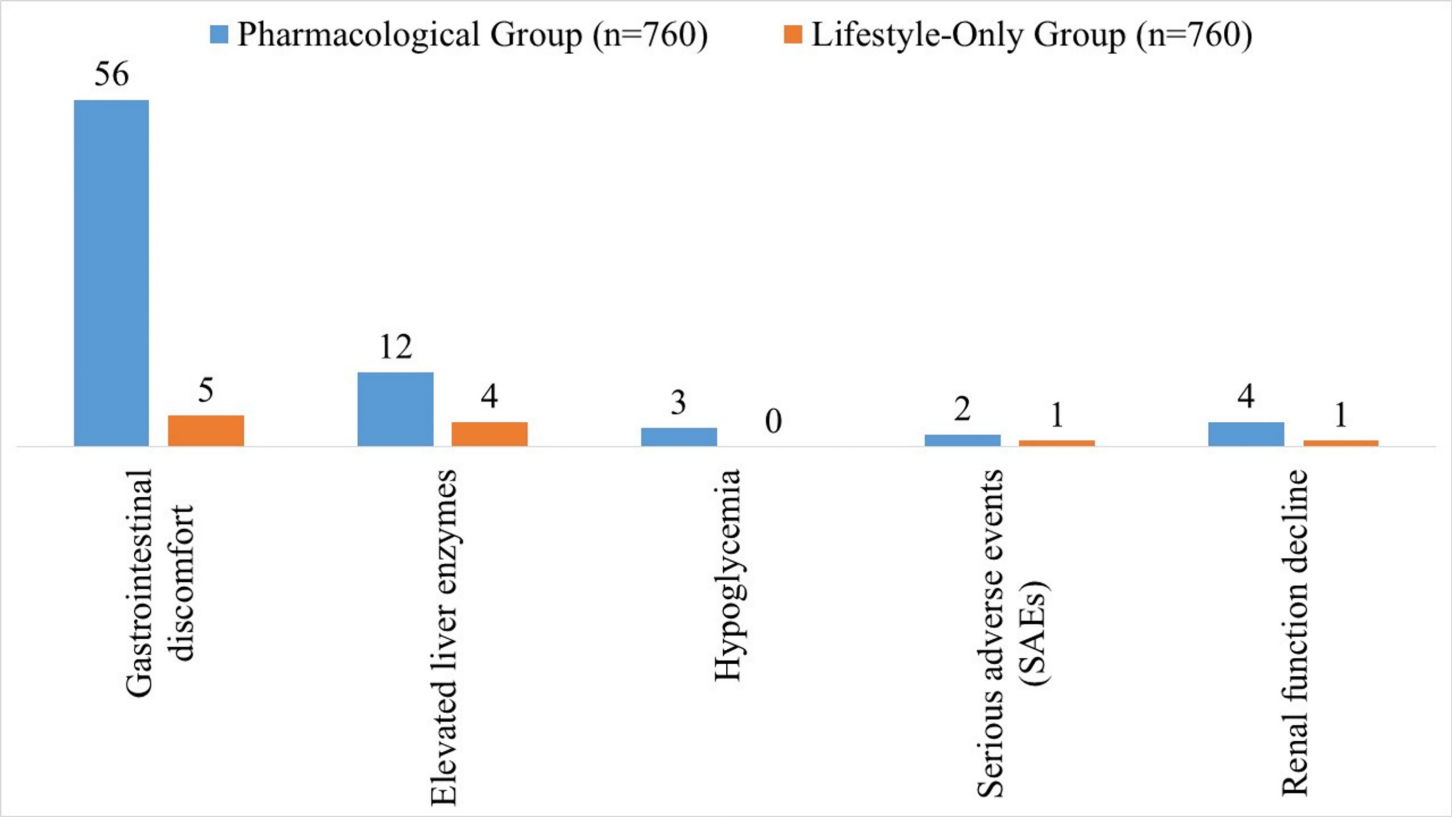
Adverse events and safety at follow-up visits

Adverse events were more common in the pharmacological group, including gastrointestinal discomfort (56 vs. 5), elevated liver enzymes (12 vs. 4), and hypoglycemia (3 vs. 0). Serious adverse events were rare (2 vs. 1), and renal function decline occurred in 4 vs. 1 participant, demonstrating an overall acceptable safety profile (Figure 3).

**Figure 4:**
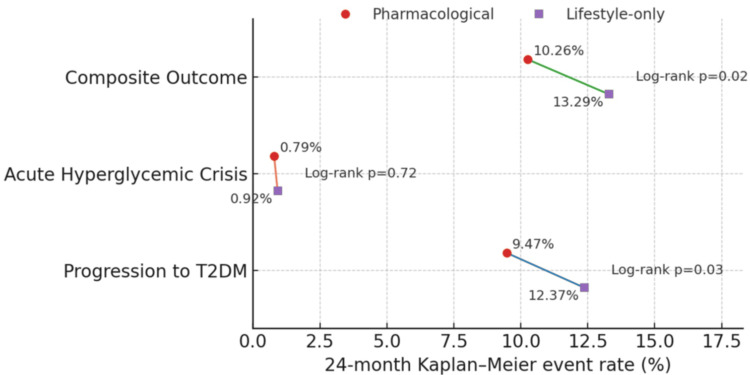
Kaplan-Meier estimated 24-month event rates by intervention Points show group rates; connecting lines highlight between-group differences. Log-rank p values shown per outcome T2DM: type 2 diabetes mellitus

The pharmacological group demonstrated lower rates of progression to T2DM compared to the lifestyle-only group, with 9.47% vs. 12.37% of participants affected (p = 0.03), as shown in Figure 4. Acute hyperglycemic crises were rare in both groups, occurring in 0.79% of the pharmacological group and 0.92% of the lifestyle group (p = 0.72). For the composite outcome of progression to T2DM or hyperglycemic crises, the pharmacological group also fared better at 10.26% vs. 13.29% in the lifestyle group (p = 0.02), indicating a modest but statistically significant advantage for pharmacological intervention in preventing adverse glycemic events.

**Figure 5:**
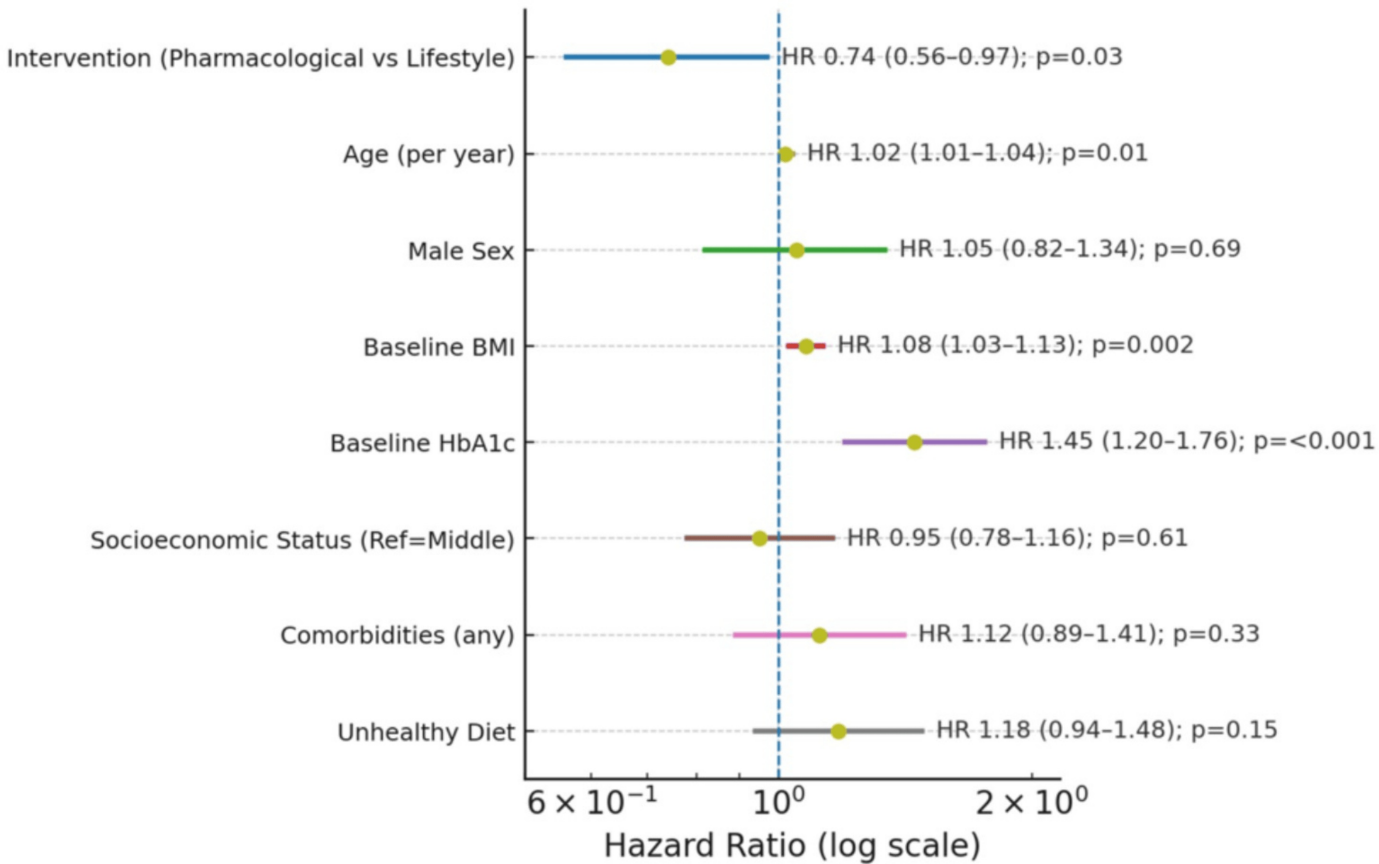
Cox proportional hazards analysis for progression to T2DM/acute crises Dots indicate HRs; horizontal lines show 95% CIs; vertical dashed line marks HR = 1.0. Cox proportional hazards regression applied; significance considered at p < 0.05 T2DM: type 2 diabetes mellitus; HRs: hazard ratios; CIs: confidence intervals; BMI: body mass index; HbA1c: glycated hemoglobin

Cox regression analysis confirmed the protective effect of pharmacological intervention, with an HR of 0.74 (95% CI: 0.56-0.97, p = 0.03) relative to lifestyle-only therapy (Figure 5). Higher age (HR: 1.02 per year, p = 0.01), baseline BMI (HR: 1.08, p = 0.002), and baseline HbA1c (HR: 1.45, p < 0.001) were significant predictors of progression. Other variables, including male sex, socioeconomic status, comorbidities, and unhealthy diet, were not statistically significant. These findings highlight that pharmacological intervention significantly reduces the risk of progression while accounting for key demographic and metabolic risk factors.

## Discussion

In this longitudinal multicenter study, both pharmacological and lifestyle-only interventions produced significant improvements in anthropometric and metabolic parameters over 24 months in adults with prediabetes. Participants in the pharmacological group experienced a mean weight reduction of 3.75 kg and a BMI decrease of 1.35 kg/m². In comparison, those in the lifestyle-only group lost 4.65 kg and 1.60 kg/m², respectively (p > 0.05). These findings align with prior research demonstrating that structured lifestyle programs are effective for weight reduction in prediabetic populations, as reported in the DPP, which has been associated with significant decreases in mean BMI, including an average reduction of 1.15 kg/m² [16]. The comparable anthropometric changes between the groups suggest that pharmacological interventions do not impair weight management and may be effectively combined with lifestyle measures.

Glycemic outcomes showed modest but significant advantages for pharmacotherapy. Mean HbA1c decreased by 0.37% in the pharmacological group versus 0.30% in the lifestyle-only group (p=0.04), and fasting plasma glucose declined by 8.6 mg/dL versus 7.1 mg/dL (p=0.03). These results are consistent with prior studies reporting that metformin therapy yields greater reductions in fasting glucose and HbA1c compared to lifestyle alone in high-risk individuals [17].

Lipid profile improvements were similar between the groups, with total cholesterol reductions of 13.5 and 12.9 mg/dL, respectively (p = 0.51), paralleling findings from the previous studies showing favorable effects of both pharmacological and lifestyle strategies on dyslipidemia [13]. Blood pressure reductions were modest and statistically nonsignificant between groups, suggesting that short-term intervention effects on hemodynamics may require longer follow-up or adjunctive strategies.

The incidence of progression to T2DM was significantly lower in the pharmacological group (9.47% vs. 12.37%, p = 0.03), with a hazard ratio of 0.74 (95% CI: 0.56-0.97), indicating a protective effect consistent with meta-analyses demonstrating a risk reduction for diabetes [18]. Acute hyperglycemic crises were rare in both groups (<1%), reflecting the overall low short-term risk in prediabetic adults but highlighting the potential benefit of early pharmacological therapy in preventing severe glycemic events. These findings reinforce reports that pharmacological therapy is especially beneficial in those with higher baseline HbA1c, a key determinant of treatment success [19]. Additionally, adherence was higher in the pharmacological group (86.05% vs. 78.68%, p = 0.004), underscoring a well-recognized challenge in sustaining lifestyle interventions over extended periods. Despite mild gastrointestinal and liver-related adverse events, pharmacotherapy demonstrated an acceptable safety profile, supporting its utility as a viable preventive strategy in prediabetes.

Study strengths and limitations

This study's major strengths include its multicenter, longitudinal design with a relatively large cohort of 1,520 prediabetic adults, ensuring diverse representation and generalizability of findings. Standardized intervention protocols, rigorous follow-up at multiple time points, and comprehensive assessment of anthropometric, glycemic, lipid, and adherence parameters enhanced data quality and internal validity. The use of both lifestyle-only and pharmacological arms allowed a direct comparative evaluation of real-world effectiveness. However, limitations include reliance on convenience sampling, which may introduce selection bias, and self-reported adherence for lifestyle interventions, which could overestimate compliance. Additionally, the follow-up duration of 24 months, while sufficient to capture early progression, may not fully reflect long-term outcomes, and rare adverse events may be underrepresented. Variability in adherence monitoring and the absence of blinding could also introduce minor performance bias, although standardized protocols across centers were implemented to minimize this effect.

## Conclusions

Both pharmacological therapy and structured lifestyle interventions led to significant improvements in anthropometric, glycemic, and lipid parameters among adults with prediabetes. Pharmacological therapy demonstrated a modest but consistent advantage in reducing the risk of progression to T2DM, with good safety and adherence profiles. Lifestyle modification alone also produced meaningful metabolic benefits, underscoring the importance of behavioral change in diabetes prevention. Collectively, these results highlight the complementary roles of pharmacological and lifestyle strategies and support the adoption of individualized, evidence-based preventive approaches to optimize long-term outcomes in high-risk prediabetic populations.
